# Laser-Based Crystallization of Chemical Solution Deposited Proton-Conducting Thin Films

**DOI:** 10.3390/ma18225235

**Published:** 2025-11-19

**Authors:** Jonas Frühling, Samuel Fink, Theodor Schneller, Christian Vedder

**Affiliations:** 1 Chair for Technology of Optical Systems (TOS), RWTH Aachen University, Steinbachstraße 15, 52074 Aachen, Germany; 2 Fraunhofer Institute for Laser Technology ILT, Steinbachstraße 15, 52074 Aachen, Germany; samuel.fink@ilt.fraunhofer.de (S.F.);; 3 Institute of Materials in Electrical Engineering and Information Technology 2 (IWE2), RWTH Aachen University, Sommerfeldstraße 24, 52074 Aachen, Germany; schneller@iwe.rwth-aachen.de

**Keywords:** barium zirconate, proton conductor, laser annealing, metal-supported, chemical solution deposition

## Abstract

This work investigates the laser-based solid-phase crystallization of wet-chemically deposited BZY (yttrium doped barium zirconate) thin films on metallic substrates. For this purpose, amorphous BZY thin films are deposited on nickel-based alloy substrates using spin coating and are then annealed using laser radiation. Different laser intensities and scanning velocities are investigated. X-ray diffraction analysis of the processed thin films shows an initial increase in crystallinity with increasing laser intensity. A further increase in laser intensity leads to the formation of secondary phases and ultimately to the melting of the substrate material. Complete crystallization of the thin films without the formation of secondary phases is achieved by applying scanning velocities of $v_{S}$ ≥ 500 mm/s. Scanning electron microscopy images of selected samples show that, especially at higher scanning velocities, crack formation can occur as a result of the annealing. In summary, laser annealing is a promising approach for the thermal post-treatment of BZY thin films in applications in metal-supported solid oxide fuel cells.

## 1. Introduction

As a result of global efforts to reduce greenhouse gas emissions, renewable energy sources (wind power, hydropower, solar power, etc.) are now the focus of global energy policy. However, solar power and wind power in particular are subject to natural fluctuations and therefore cannot provide a constant energy supply. To bridge these fluctuations, there is a need for cost-effective long-term energy storage solutions. The power-to-gas (P2G) approach for seasonal storage in the form of chemical energy (e.g., hydrogen, methane or ammonia) in the megawatt to gigawatt range represents a promising solution [1,2,3]. Suitable electrochemical converters play a central role in the large-scale, cost-efficient implementation of the P2G approach. Solid oxide fuel cells (SOFCs) and solid oxide electrolyzer cells (SOECs) are subjects of current research in this field [4,5,6,7,8,9,10,11,12]. Especially, cells based on proton-conducting electrolytes received a lot of attention in this context and are promising technologies in this regard [13,14,15,16,17,18,19].

The use of proton-conducting perovskites as electrolytes enables lower operating temperatures of approx. 400–600 °C compared to conventional oxide ion-conducting electrolytes, such as yttrium-stabilized zirconium oxide (YSZ) of approx. 800–1000 °C [20,21]. Furthermore, it improves cyclability, i.e., the repeated heating and cooling of the cell, and prevents anode oxidation [22,23,24].

The most common proton-conducting oxides are ABO_3_ perovskites. Zr [25,26], Ce [27,28] or a mixture of both [29,30] are generally used as the A-site cation [31]. Y is usually used as the B-site cation. Yb [32,33] or a mixture of Y and Yb [34] have also been reported.

Due to its high ionic bulk conductivity and greater chemical stability compared to yttrium-doped barium cerate (BCY), yttrium-doped barium zirconate (BZY) is the most widely investigated perovskite proton conductor. However, in real-world applications, proton conductivity is limited by the crystallite size due to the high resistance of the grain boundaries [35,36]. Due to the poor sinterability of the material, very high temperatures (up to 2200 °C) and holding times are necessary to achieve large crystallite sizes [37].

This leads to challenges in the manufacturing process of BZY thin films. This is especially so in the case of metal-supported cells, which have several advantages over conventional anode-supported cells, such as greater mechanical resistance during thermal cycling and potentially greater cost efficiency [38,39,40]. Applying the necessary temperatures, e.g., in long-lasting oven sintering processes, can lead to the formation of undesirable secondary phases due to interactions between the substrate and the functional layers, such as oxidation of the substrate material and barium evaporation from the electrolyte layer [41,42]. The presence of such secondary phases, including possible sintering aids, often leads to reduced performance [41,42,43,44].

In addition to various vacuum-based processes such as atomic layer deposition (ALD) [45], magnetron sputtering [46,47], electron beam evaporation (EBE) [48] and pulsed laser deposition (PLD) [26,49,50], chemical solution deposition (CSD) [51] is an attractive technology for the production of BZY thin films. In addition to eliminating the need for complex vacuum technology, it enables precise adjustment of the stoichiometry, deposition of homogeneous layer thicknesses and comparatively low equipment costs [52]. However, at approximately 10^−3^–10^−7^ S/cm [52], the proton conductivities achieved are still several orders of magnitude lower than those of bulk material [37,53,54,55,56,57,58,59].

Laser annealing represents an innovative approach for thermal post-treatment of BZY thin films deposited by CSD in order to increase the crystallite size and thus the proton conductivity. The main advantages are the high annealing temperatures, which can be achieved while simultaneously significantly reducing the temperature holding times. This is intended to reduce the thermal load on the substrate material, prevent the formation of secondary phases and produce potentially larger crystallite sizes than with conventional methods. While first investigations on the laser annealing of BZY thin films deposited by magnetron sputtering on ceramic substrates [47] and the laser annealing of other oxide ceramic thin films (e.g., lead zirconate titanate (PZT)) [60] deposited by CSD are available, the laser annealing of BZY thin films deposited by CSD on metallic substrates is investigated for the first time in this work.

The overall objective of this paper is to conduct a fundamental investigation into the feasibility of laser-based solid-phase crystallization of BZY thin films deposited by CSD on metallic substrates. To this end, the influence of laser intensity and scanning velocity on the crystallographic properties and microstructure of the processed films is being investigated.

## 2. Materials and Methods

### 2.1. Sample Preparation and Precursor Chemistry

The BZY thin films investigated in this study were deposited using CSD. For the synthesis of the BZY precursor solution, a metallo-organic decomposition (MOD) approach has been chosen, which has the advantage of being less humidity-sensitive and of being chemically stable over several weeks. An acceptor dopant concentration of 10% Y corresponding to a composition BaZr_0.9_Y_0.1_O_3−δ_ (BZY10) was applied. Hence, in a typical batch, 50 mL of the BZY10 precursor solution was prepared by refluxing a mixture of 2.960 g barium carbonate (Puratronic^®^, 99.997%, Alfa Aesar GmbH & Co KG, Karlsruhe, Germany), 6590 g zirconium (IV) 2,4-pentanedionate (99.9%, Alfa Aesar GmbH & Co KG, Karlsruhe, Germany) and 0.599 g yttrium(III) 2,4-pentanedionate hydrate (Alfa Aesar, 99.9%, Alfa Aesar GmbH & Co KG, Karlsruhe, Germany), dispersed in 32 mL of a mixture of propionic acid and propionic acid anhydride (volume ratio = 4:1) in a 100 mL round bottom flask. The propionic acid anhydride was added to remove the released water of the reaction. After approx. 3 h at 140 °C, a clear brownish solution was obtained and the final concentration of 0.3 M was achieved after filling up with propionic acid by means of a volumetric flask.

A sheet material made of the Hastelloy^®^-alloy C276 (High Tech Alloys Sonderwerkstoffe GmbH, Hilden, Germany) was used as a substrate. The square substrates had an initial length of 25.4 mm and a thickness of 2 mm. After cleaning with ethanol in an ultrasonic bath for 5 min (Fisher Scientific International, Inc., Hampton, NH, USA), the substrates were dried on a hot plate at 100 °C for 1 min. Afterwards, the samples were coated using a POLOS SPIN150i spin coater (SPS-Europe B.V., Putten, The Netherlands). A total of 175 µL of the precursor solution was deposited onto the substrate, followed by a spin coating process, starting with 3000 rpm for 30 s followed by 500 rpm for 5 s. After the spin coating process, the samples were pyrolyzed on a PZ 28-3T hot plate (Harry Gestigkeit GmbH, Düsseldorf, Germany) at 400 °C for 5 min to remove any organic residue in the thin films. This procedure was conducted three times to reach the desired film thickness of about 200 nm.

### 2.2. Laser Annealing

**Figure 1:**
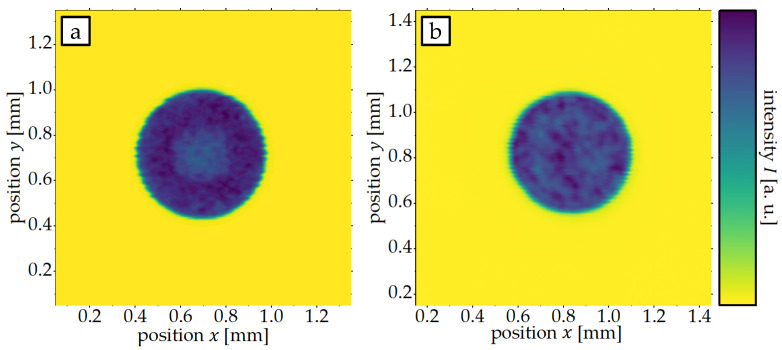
Relative intensity distribution of the laser radiation in the focus plane for LS1 (**a**) and LS2 (**b**).

The laser annealing of the samples was carried out using two continuous wave (cw) fiber lasers: an IPG YLR-200-MM-AC-Y11 (IPG Photonics GmbH & Co. KG, Burbach, Germany) with a wavelength of 1070 nm, hereinafter referred to as laser system 1 (LS1) with a nominal maximum output power of 200 W and a laser, and an SP-2000-C-W-025-10-PIQ-019-001-000 (SPI Lasers UK Ltd., Rugby, UK) with a wavelength of 1075 nm, hereinafter referred to as laser system 2 (LS2) with a nominal maximum output power of 2000 W. The relative intensity distribution of the laser beam in the focal plane exhibits a top-hat intensity distribution (homogenous intensity across the beam profile) for both laser systems and is shown in Figure 1. The laser beam diameters $w_{L}(z=0)$ in the focal plane are 566 µm for LS1 and 546 µm for LS2.

Within the scope of the presented investigations, the laser intensity $I_{L}$ and scanning speed $v_{S}$ are varied. The laser intensity is calculated from the laser power $P_{L}$ and the irradiated area $A_{L}$ (1) and is adjusted by modifying the laser power.

(1) $$I_{L}=\frac{P_{L}}{A_{L}}=\frac{P_{L}}{\pi \cdot {\left(\frac{w_{L}(z=0)}{2}\right)}^{2}}$$

**Figure 2:**
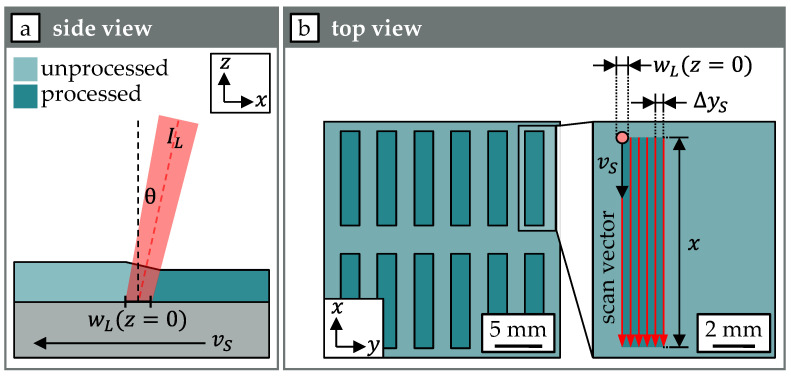
Illustration of the processing strategy and process parameters in side view (**a**) and top view (**b**).

On one sample, twelve areas were processed with different processing parameters. Each area was irradiated using six unidirectional scan vectors with a fixed length x = 10 mm. The distance between the individual scan vectors was fixed for all experiments at ${∆y}_{S}$ = 400 µm. To prevent damage to the laser beam sources as a result of direct back reflection of the laser beam from the sample surface, processing was carried out at the edge of the scan field with incident angles θ larger than 16.8°. A schematic illustration of this process strategy is shown in Figure 2.

### 2.3. Sample Characterization

The processed areas were analyzed by X-ray diffraction (XRD) using a D8 ADVANCE (Bruker AXS Holdings, Inc., Billerica, MA, USA). Cu Kα radiation at 1.5406 Å was used and the measurements were carried out in Theta-2Theta-geometry. To quantify the crystallinity, the integrated peak intensity of the reflections assigned to the crystalline BZY phase was used (2):

(2) $$I_{BZY}=I_{BZY(110)}+I_{BZY(211)}+I_{BZY(220)}+I_{BZY(310)}$$

For the investigation of the crystallite size $d_{c}$, a structural refinement was performed using the software TOPAS-64 V7.13 (Bruker AXS Holdings, Inc., Billerica, MA, USA).

Top view SEM (Apreo 2C, Thermo Fisher Scientific Inc., Waltham, MA, USA) images were made from selected processed areas to investigate the density and possible defects. Therefore, a T1 detector and an Everhart-Thornley detector (ETD) were used. The T1 detector records the backscattered electrons. It is suitable for displaying material contrast. The ETD records the secondary electrons and is suitable for displaying topographical contrast.

## 3. Results and Discussion

### 3.1. Influence of Laser Intensity

**Figure 3:**
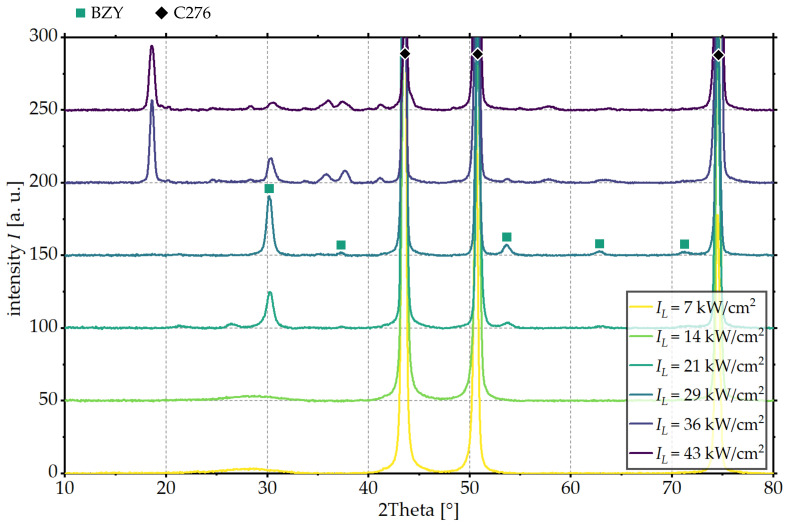
XRD patterns of fields processed with varying laser intensity $I_{L}$ and a scanning velocity of $v_{S}$ = 50 mm/s using LS1.

To evaluate the influence of the laser intensity on the phase composition of the BZY thin films, XRD measurements of the laser processed area were carried out. The XRD patterns for BZY thin films processed with different laser intensities and a constant scanning velocity of $v_{S}$ = 50 mm/s are presented in Figure 3.

For all fields examined, the reflections with the highest intensity were assigned to the substrate material C276. For small laser intensities of $I_{L}$ = 7 kW/cm^2^ and $I_{L}$ = 14 kW/cm^2^, a broadened reflection associated with the amorphous BZY phase is observed at about 28.5°. For laser intensities above $I_{L}$ = 21 kW/cm^2^, reflections at 30.2°, 37.3°, 53.6°, 62.7° and 71.2° associated with the crystalline BZY phase are identified. In addition, a reflection at 2Theta = 26.5° is observed, which is no longer present at higher laser intensities. However, a clear identification of these reflections is not possible using XRD measurements alone. Potential phases are, for example, metallic Ba or Y, which could both oxidize when higher laser intensities are applied.

Initially, the intensity of the reflections assigned to the crystalline BZY phase increases with increasing laser intensity. For laser intensities of $I_{L}$ ≥ 36 kW/cm^2^, a decrease in the reflections’ intensity assigned to the crystalline BZY phase is observed. This is accompanied by the appearance of further reflections at 18.6°, 35.8°, 37.7°, 57.8° and 71.2°, which are attributed to the formation of secondary phases. It is not possible to clearly identify these phases using XRD measurements alone. Various oxides (NiO, Cr_2_O_3_ and Cr_2_NiO_4_) of the substrate material are considered as possible phases. Also, the chromium might have diffused from the substrate into the BZY thin film and formed secondary phases with elements of the coating material. To clearly identify the observed secondary phases, further material investigations are necessary. These could include X-ray photoelectron spectroscopy (XPS) measurements or high-resolution EDX (energy-dispersive X-ray spectroscopy) measurements using transmission electron microscopy (TEM), for example. In summary, for a scanning velocity of 50 mm/s, a crystalline BZY phase can be achieved without the formation of secondary phases when using a laser intensity of 29 kW/cm^2^. A further increase in laser intensity leads to degradation of the crystalline BZY phase.

### 3.2. Influence of Scanning Velocity

**Figure 4:**
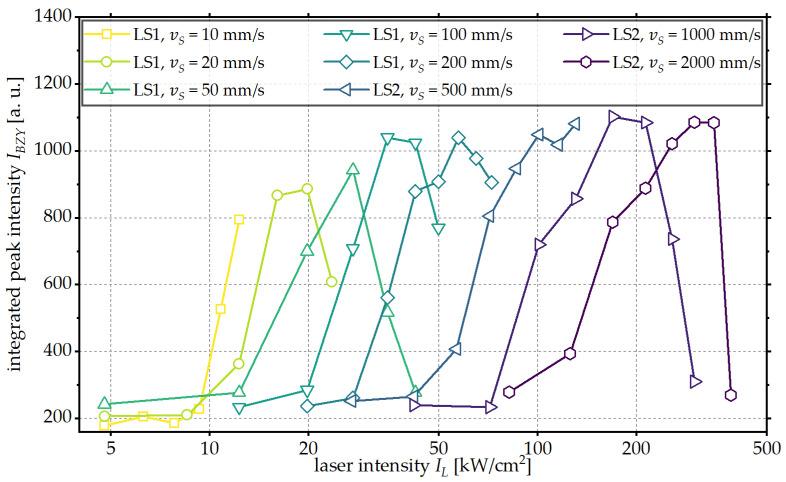
Integrated peak intensity $I_{BZY}$ of the BZY reflections as a function of the laser intensity $I_{L}$ for different scanning velocities $v_{S}$.

To evaluate the influence of the scanning velocity, the integrated peak intensity of the reflections assigned to the crystalline BZY is used as a measure of the crystallinity of the processed BZY thin films. This is shown in Figure 4 as a function of the logarithmic irradiation intensity for the scanning velocities investigated.

A similar trend is observed for all scanning velocities investigated. An increase in the laser intensity initially results in an increase in the integrated peak intensity and thus in an increase in the crystallinity of the BZY phase. After reaching the maximum integrated peak intensity, a steep decrease in the integrated peak intensity is observed with further increasing laser intensity. This is attributed to the degradation of the crystalline BZY phase and eventually the melting of the substrate material, which is observed when the energy input into the sample is too high.

With increased scanning velocity, higher laser intensities are required for the crystallization of the thin film. For example, at the lowest investigated scanning velocity of 10 mm/s, first reflections assigned to a crystalline BZY phase can be observed for a laser intensity of 10.8 kW/cm^2^, while, at a scanning velocity of 1000 mm/s, a laser intensity of 100.8 kW/cm^2^ is required to achieve the first reflections.

In addition, higher maximal integrated peak intensities are calculated. One possible explanation is that the longer interaction times at lower scanning velocities lead to the degradation of the BZY thin film as a result of diffusion prior to the completed conversion to the crystalline phase, as observed in Figure 3 for laser intensities of $I_{L}$ ≥ 36 kW/cm^2^.This could be attributed to the diffusion of elements from the substrate material into the thin film. This effect is also described in the literature, for example, for the diffusion of Ni and Fe [42]. Complete crystallization of the thin film without the formation of secondary phases would therefore not be possible at lower scanning velocities or higher interaction times, respectively.

**Figure 5:**
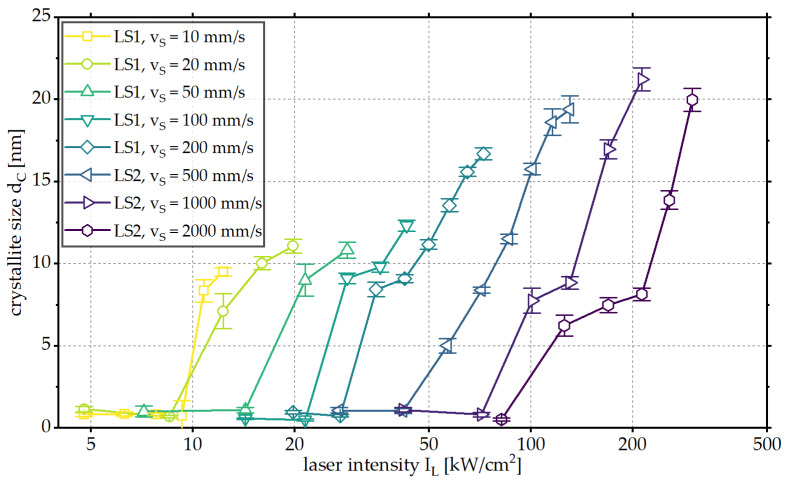
Crystallite size $d_{c}$ of the BZY reflections as a function of the laser intensity $I_{L}$ for different scanning velocities $v_{S}$.

In addition to the crystallinity of the thin film, the achieved crystallite size of BZY within the processed areas was investigated. The results are presented in Figure 5 for the used scanning velocities and laser intensities. Only laser intensities at which no degradation of the BZY phase had been found are considered.

An increase in the laser intensity leads to an increase in crystallite size for all scanning velocities investigated up to the degradation of the BZY thin film. With increasing scanning velocity, an increase in the achievable maximum crystallite sizes from $d_{C}$ = 9.5 nm for $v_{S}$ = 10 mm/s to $d_{C}$ = 21.2 nm for $v_{S}$ = 1000 mm/s is observed.

**Figure 6:**
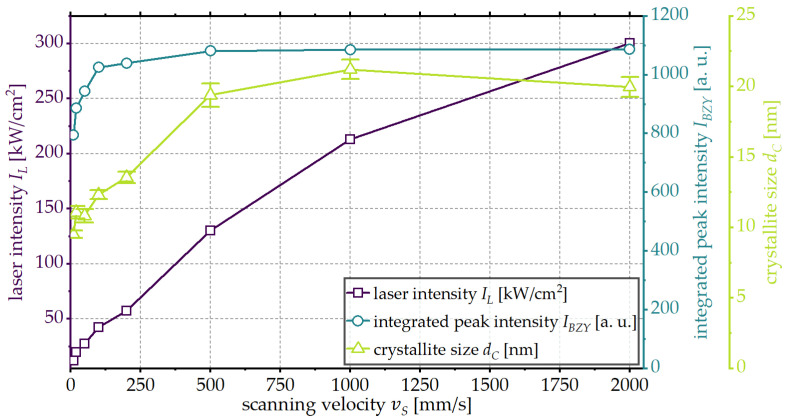
Integrated peak intensity $I_{BZY}$, crystallite size $d_{C}$ and laser intensity $I_{L}$ at which the highest integrated peak intensity was achieved in dependence of the investigated scanning velocities.

In order to compare the results, the laser processed areas with the highest integrated peak intensity and thus the highest crystallinity are investigated. The integrated peak intensity and the crystallite size, as well as the according laser intensity used for processing, are shown in Figure 6.

The laser intensity used to process the areas with the highest integrated peak intensity increases with increasing scanning velocity. For scanning velocities of $v_{S}$ ≥ 250, this increase is approximately linear. The maximal achievable integrated peak intensity initially increases up to a scanning velocity of $v_{S}$ = 500 mm/s and stays constant at higher scanning velocities, which is interpreted as complete crystallization of the BZY thin film. For scanning velocities of $v_{S}$ < 500 mm/s, complete crystallization of the BZY thin film is therefore not possible without degradation of the BZY phase and/or melting of the substrate material. The influence of the scanning velocity on the maximal achievable crystallite size shows a similar trend. Up to a scanning speed of $v_{S}$ = 500 mm/s, an increase in scanning velocity leads to an increase in the maximal crystallite size. For a further increase, a crystallite size that is constant within the margin of error is observed.

One possible explanation for the smaller crystallite sizes at scanning velocities of $v_{S}$ < 500 mm/s is the degradation of the BZY phase prior to complete crystallization, as discussed above. Another possible explanation is that higher scanning velocities and thus shorter interaction times allow higher temperatures to be achieved without the degradation of the BZY phase.

**Figure 7:**
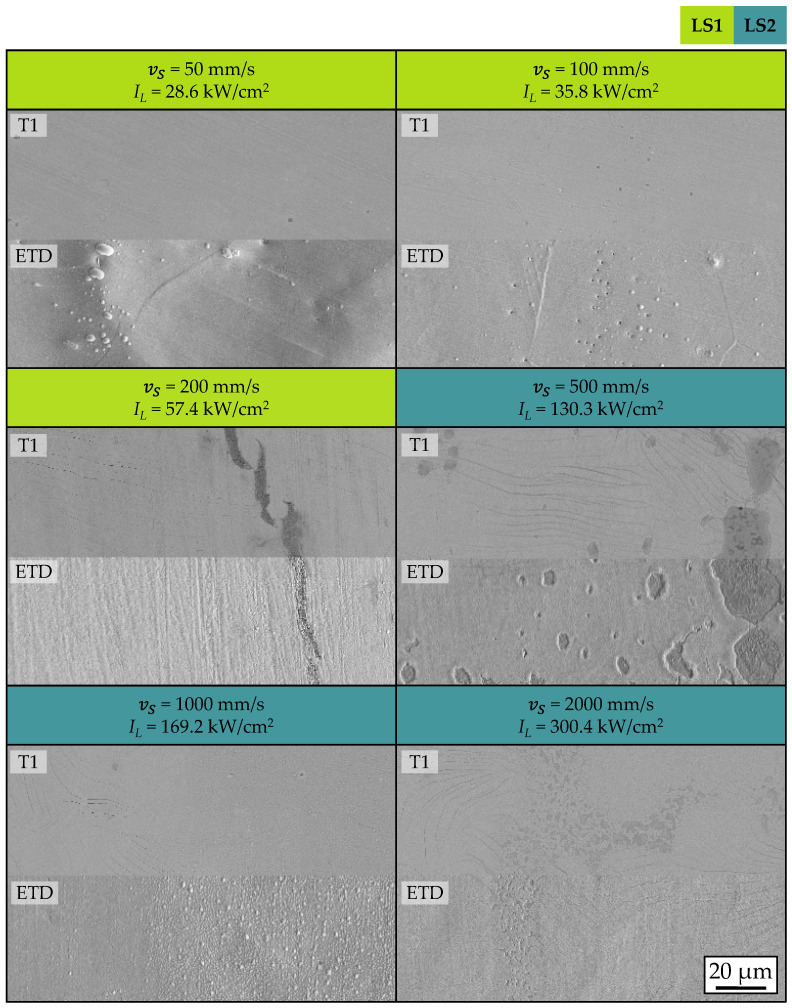
Top view SEM images of processed BZY thin films with maximal integrated peak intensity for the investigated scanning velocities. The images were acquired using a T1 detector and an ETD (Everhart-Thornley detector).

In addition to the crystallinity and crystallite size of the processed thin films, their microstructure was investigated using scanning electron microscopy (SEM). A T1 detector and an ETD were used for this purpose. The top view images of the field with the highest peak intensity for scanning velocities from 50 mm/s to 2000 mm/s are shown in Figure 7.

**Figure 8:**
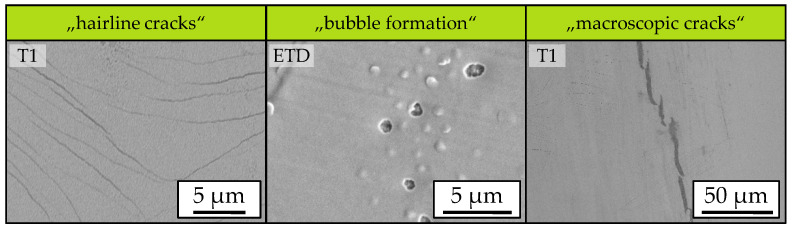
Examples of defect types observed using top view SEM images.

At scanning velocities of $v_{S}$ = 200 mm/s and above, cracks are observed within the BZY thin film. In addition to these macroscopic cracks, other types of defects are also observed. These include fine hairline cracks with a width of a few hundred nanometres. Like the macroscopic cracks, these occur at scanning velocities $v_{S}$ ≥ 200 mm/s. Furthermore, bubble formation within the thin film is detected for all parameters examined. Open pores are also observed in the SEM image of the thin film processed at a scanning velocity of $v_{S}$ = 500 mm/s, which are attributed to spalling as a result of bubble formation. Examples of the observed defect types are shown in Figure 8.

The occurrence of the observed crack formation is attributed to the CTE (coefficient of thermal expansion) difference between the coating material BZY ($\alpha_{BZY}$ = 8.8 × 10^−6^ 1/°C [61]) and the substrate material C276 ($\alpha_{C276}$ = 11.2 × 10^−6^ 1/°C) of 2.4 × 10^−6^ 1/°C. One explanation for the influence of the scanning velocity on crack formation is that higher temperature gradients along the substrate surface are expected when using higher scanning velocities. These lead to thermally induced stress and, as a result, to crack formation.

Since the gas tightness of the electrolyte layer is necessary for the application in an SOFC, further investigations should focus on preventing the observed defect formation.

## 4. Conclusions

In this work, a novel laser-based approach for the solid-phase crystallization of CSD-derived BZY thin films on metallic substrates is presented. Due to the short interaction times, this approach enables the complete crystallization of BZY thin films on metallic substrates at scanning velocities of $v_{S}$ ≥ 500 mm/s, while avoiding undesirable secondary phases. In addition, increasing the scanning velocity leads to an increase in the maximal achievable crystallite size up to 21.2 nm for $v_{S}$ = 1000 mm/s and $I_{L}$ = 213 kW/cm^2^. However, the CTE difference between the substrate material C276 and the coating material BZY leads to the formation of cracks within the coating. This occurs at scanning velocities of ≥200 mm/s. At scanning velocities < 200 mm/s, a defect-free coating is produced, although the crystallization process is not completed. Accordingly, there is a conflict of objectives between the degree of crystallinity and the crystallite size on one side and a defect-free coating and the avoidance of crack formation on the other. Possible approaches to avoiding crack formation include preheating during annealing or adjusting the coating system. Next possible steps include investigating the ionic conductivity of the crystallized coating and further developing the process with a focus on achieving a fully crystallized defect-free coating and further increasing the crystallite size.

## Data Availability

The original contributions presented in the study are included in the article, further inquiries can be directed to the corresponding author.

## Abbreviations

The following abbreviations are used in this manuscript:

ALD	Atomic Layer Deposition
BCY	Yttrium-Doped Barium Cerate
BZY	Yttrium-Doped Barium Zirconate
CSD	Chemical Solution Deposition
CTE	Coefficient of Thermal Extension
cw	Continuous Wave
EBE	Electron Beam Evaporation
EDX	Energy Dispersive X-ray Spectroscopy
ETD	Everhart-Thornley Detector
LS1	Laser System 1
LS2	Laser System 2
MOD	Metallo-Organic Decomposition
PG	Power to Gas
PLD	Pulsed Laser Deposition
PZT	Lead Zirconate Titanate
SEM	Scanning Electron Microscopy
SOEC	Solid Oxide Electrolyzer Cell
SOFC	Solid Oxide Fuel Cell
TEM	Transmission Electron Microscopy
XPS	X-Ray Photoelectron Spectroscopy
XRD	X-Ray Diffraction
YSZ	Yttrium-Stabilized Zirconium Oxide
